# Identification and Characterization of an Affimer Affinity Reagent for the Detection of the cAMP Sensor, EPAC1

**DOI:** 10.3390/cells10092307

**Published:** 2021-09-03

**Authors:** Hanna K. Buist, Urszula Luchowska-Stańska, Boy van Basten, Jessica Valli, Brian O. Smith, George S. Baillie, Colin Rickman, Bryon Ricketts, Alex Davidson, Ryan Hannam, Joanne Sunderland, Stephen J. Yarwood

**Affiliations:** 1 Institute of Biological Chemistry, Biophysics and Bioengineering, School of Engineering and Physical Sciences, Heriot-Watt University, Edinburgh Campus, Edinburgh EH14 4AS, UK; hannabuist@hotmail.com (H.K.B.); ul2@hw.ac.uk (U.L.-S.); b.v.basten@gmail.com (B.v.B.); j.valli@hw.ac.uk (J.V.); c.rickman@hw.ac.uk (C.R.); 2 Institute of Molecular Cell and System Biology, College of Medical, Veterinary & Life Sciences, University of Glasgow, Glasgow G12 8QQ, UK; brian.smith@glasgow.ac.uk; 3 Institute of Cardiovascular & Medical Science, College of Medical, Veterinary & Life Sciences, University of Glasgow, Glasgow G12 8QQ, UK; george.baillie@glasgow.ac.uk; 4 Avacta Life Sciences, Unit 20, Ash Way, Thorp Arch Estate & Retail Park, Wetherby LS23 7FA, UK; bryon.ricketts@avacta.com (B.R.); alex.davidson@avacta.com (A.D.); ryan.hannam@avacta.com (R.H.); joanne.sunderland@avacta.com (J.S.)

**Keywords:** EPAC1, cyclic AMP, affimer, microscopy, protein interactions, phage display

## Abstract

An exchange protein directly activated by cAMP 1 (EPAC1) is an intracellular sensor for cAMP that is involved in a wide variety of cellular and physiological processes in health and disease. However, reagents are lacking to study its association with intracellular cAMP nanodomains. Here, we use non-antibody Affimer protein scaffolds to develop isoform-selective protein binders of EPAC1. Phage-display screens were carried out against purified, biotinylated human recombinant EPAC1ΔDEP protein (amino acids 149–811), which identified five potential EPAC1-selective Affimer binders. Dot blots and indirect ELISA assays were next used to identify Affimer 780A as the top EPAC1 binder. Mutagenesis studies further revealed a potential interaction site for 780A within the EPAC1 cyclic nucleotide binding domain (CNBD). In addition, 780A was shown to co-precipitate EPAC1 from transfected cells and co-localize with both wild-type EPAC1 and a mis-targeting mutant of EPAC1(K212R), predominantly in perinuclear and cytosolic regions of cells, respectively. As a novel EPAC1-selective binder, 780A therefore has the potential to be used in future studies to further understand compartmentalization of the cAMP-EPAC1 signaling system.

## 1. Introduction

The universal second messenger, 3′,5′-cyclic adenosine monophosphate (cAMP), was the first to be discovered in eukaryotic cells in 1958 by Earl Sutherland and colleagues [1]. The cAMP signaling pathway is now considered to be a paradigm of intracellular signaling and is involved in a wide variety of cellular and physiological processes [2,3,4,5]. Activation of Gs protein-coupled receptors (GsPCRs) promotes activation of intracellular adenylyl cyclases (AC), leading to the generation of cAMP inside the cell from ATP. Signal termination occurs through the hydrolytic action of cAMP phosphodiesterase (PDE) enzymes and receptor desensitization. There are nine membrane-bound, and one soluble, isoforms of AC, whereas the PDE superfamily is made up of 21 genes, grouped into 11 diverse families (PDEs 1–11) [6,7].

The downstream signaling effects in response to cAMP production were originally attributed to activation of protein kinase A (PKA) isoforms [8]. For example, gene transcription induced by cAMP is mediated through PKA phosphorylation of the cAMP response element-binding protein (CREB) transcription factor, amongst others [9]. Subsequently, a new cAMP sensor, exchange protein directly activated by cAMP (EPAC) was identified, showing that the activation of the small GTPase, Rap1, by cAMP occurred independently of PKA [10,11]. Cyclic nucleotide-gated ion channels (HCN) and Popeye domain containing (POPDC) gene family are two additional recently discovered cAMP effectors [12,13,14]. As effector proteins, PKA and EPAC are known to be anchored to specific intracellular sites by scaffold proteins, which act to maintain compartmentalization of cAMP signaling by anchoring PDEs adjacent to target effectors and, consequently, regulate the ability of cAMP to initiate downstream signaling events at discrete subcellular compartments or nanodomains [15].

Evidence is emerging that compartmentalization of the two EPAC isoforms, EPAC1 and EPAC2, is key to tailoring their effector functions. For example, in vascular endothelial cells (VECs), VE-cadherin-based adhesion and cell permeability are mediated by PDE4D regulation of EPAC1 signaling complexes [16]. In the first study to investigate the subcellular localization of EPAC1, two distinct populations of EPAC1 were observed [17] in EPAC1-GFP transfected COS7 cells, where EPAC1 was localized mainly in and around the nucleus, along the nuclear membrane, but also in punctate structures identified to be the mitochondria in these cells [17]. An increase in levels of EPAC1 at the nuclear membrane was observed in these cells following stimulation with forskolin and rolipram [17]. Importantly, this was the first study to show that the EPAC1 membrane association was due to the EPAC1 Disheveled-EGL-plekstrin homology (DEP) domain [17]. From this study, it was further determined that the localization of EPAC1 was cell cycle dependent, with EPAC1 being mainly associated with the nuclear membrane during metaphase, and then further associated with the mitotic spindle and centrosomes following the breakdown of the nuclear envelope [17]. The localization of EPAC1 to the perinuclear region of cells has also been seen in several other cell lines, including COS1, human embryonic kidney (HEK293) cells and cancer cell lines, such as the human epidermal carcinoma (A431) and the human breast cancer (MCF7) cells [17,18,19,20,21,22,23]. This is consistent with Rap1 fluorescence resonance energy transfer (FRET) activation studies that demonstrate activation of Rap1 in this region following elevations in intracellular cAMP [21]. Inactive EPAC1 appears to be tethered to the nuclear envelope through interactions with the scaffold protein, Ran binding protein 2 (RanBP2) [24,25], which is a component of the nuclear pore complex, although the activation/inactivation cycle of EPAC1 at the nuclear/perinuclear region remains to be determined.

EPAC1 has also been observed, via confocal microscopy, total internal reflection fluorescence (TIRF) and FRET analysis, to translocate to the plasma membrane (PM) in HEK293 cells, at early time points following cAMP stimulation [22]. Again, the EPAC1 DEP domain was found to be essential for EPAC1 translocation, but in cooperation with the catalytic region [22], through interactions with the negatively charged phospholipid, phosphatidic acid (PA) [26]. This results in the tethering of EPAC1 to the PM, allowing for Rap1 activation and facilitating cell adhesion [26]. In addition, EPAC1 recruitment to the PM can occur independently of the DEP domain via interaction of the first 49 residues at the N-terminus of EPAC1 with the ezrin-radixin-moesin (ERM) family of actin-binding proteins [27]. When active, ERM proteins, such as ezrin, can bind to EPAC1 and recruit the protein to PM in clusters, working with DEP-domain mediated translocation to control cell adhesion [27]. EPAC2 has also been observed to localize at the PM, through interactions with PM-bound Ras, leading to localized Rap1 activation [23,28,29].

The distinct distributions of the EPAC1 and EPAC2 isoforms have also been studied in adult mouse cardiomyocytes using a novel fluorescent EPAC ligand, 8-[PharosΦ-cAMP]-2′-O-methyladenosine-3′,5′-cyclic monophosphate (Φ-O-Me-cAMP) [30]. In these studies, Φ-O-Me-cAMP offered an advantage over EPAC1- and EPAC2-selective antibodies, which show a lack of specificity in immunolocalization studies [30]. With Φ-O-Me-cAMP it was observed that endogenous EPAC2 levels were concentrated at T-tubules while endogenous EPAC1 was concentrated at perinuclear regions [30]. These different localizations of EPAC1 and EPAC2 are no doubt important for their isoform-specific regulation in cells and consequently their diverse physiological functions.

Despite the emergence of Φ-O-Me-cAMP, there are still very few tools available to study EPAC1 localization in cells beyond transfection with labelled isoform. There is a commercially available monoclonal antibody (Ab), 5D3, which was first developed by the Bos laboratory but is limited in its usefulness because it acts as an allosteric agonist towards EPAC1 [31]. 5D3 specifically activates EPAC1 by binding to an epitope in the EPAC1 CNBD that is normally hidden through interactions with the catalytic region [31]. In addition, an EPAC2-specific monoclonal antibody, 5B1, has also been developed, with its epitope shown to be in a similar area of the EPAC2 CNBD as 5D3 [31]. Whereas 5D3 can be used for immunoprecipitation and immunofluorescence studies, 5B1 is unable to precipitate EPAC2 from cells [31]. Furthermore, in a study by Parnell et al., it was confirmed through IP studies that the 5D3 Ab selectively recognized the active conformation of EPAC1 in HEK293T cells transfected with FLAG tagged EPAC1, as well as the inactive form of the protein [23]. The companies Abcam and Santa Cruz both sell EPAC1- and EPAC2-selective monoclonal and polyclonal antibodies, which have been used in several different studies. For example, a recombinant anti-EPAC1 antibody from Abcam (Cat. no. ab109415) has been used in Western blotting to detect EPAC1 levels in human retinal endothelial cells (RECs) following EPAC1-agonist treatment [32]. While antibodies can be used for immunocytochemistry (ICC) or immunohistochemistry (IHC), current EPAC antibodies may not be useful for immunolocalization studies due to non-specific interactions; hence the mouse monoclonal EPAC1-A5 (Santa Cruz), goat polyclonal EPAC1-N16 (Abcam) and rabbit polyclonal anti-EPAC1 (Abcam) antibodies all gave a strong positive signal in immunocytochemistry of double knockout EPAC1 and EPAC2 mouse cardiomyocytes [30]. Thus, the current available antibodies for ICC/IHC may not be specific enough for definite localization of EPAC isoforms in cells [30].

An increasingly utilized alternative to antibody-based reagents are engineered proteins such as the peptide aptamer scaffolds, or Affimers, developed by Avacta Life Sciences (Wetherby, United Kingdom). These are small (12–15 kDa; 2–4 nm), monomeric, single-domain recombinant proteins of ~98 amino acids [33]. Affimers are selected from a library in which two loops between adjacent pairs of β-strands at one end of the domain each contain a variable sequence of nine amino acids [33,34]. These two loops typically constitute the site by which they interact with their targets. Besides being small, Affimers have several other advantages over antibodies. They are structurally stable, even under the harshest conditions, and lack disulfide bridges. Affimers do not require post-translational modification, nor do they have cysteine residues, allowing efficient expression in E.coli. In addition, they have excellent thermal and chemical stabilities with a pH range of 2–13 and a melting temperature of 101 °C. In addition, they can be easily conjugated with reporter tags or drugs [33]. Furthermore, the two variable loops provide versatile design opportunities as the two insert sequences can be randomized, allowing for identification of Affimers with desired affinity and specificity for specific targets that can be quickly generated using an established phage-display library (> 6 × 10^10^) [33].

In this study, we used the non-antibody Affimer technology to develop a selective probe that can recognize the cAMP sensor, EPAC1, for the purpose of biochemical and microscopy studies. Here, we describe the identification of EPAC1-selective Affimers from phage-display screens, their characterization in protein binding assays, further identification of an Affimer interaction site in the CNBD of EPAC1 and proof-of-principle use of an EPAC1 Affimer binder in co-immunoprecipitation and co-localization studies in transfected cells.

## 2. Materials and Methods

### 2.1. Materials

Acrylamide/Bis-acrylamide, 30% (*v/v*) solution, Ammonium-15N chloride, Ampicillin sodium salt, Adenosine 3′,5′-cyclic monophosphate (cAMP), Adenosine 5′-triphosphate disodium salt hydrate (ATP) 3,3′,5,5′-Tetramethylbenzidine Liquid Substrate, Benzamidine Sepharose 4Fast Flow (High sub), cOmplete™ Protease inhibitor cocktail, Forskolin, Helmanex™ III, ISOGRO^®^-15N Powder, Kanamycin sulfate, L-Glutathione reduced, N, N, N′, N′-tetramethylethylenediamine (TEMED), Pefabloc^®^ SC, Poly-D-Lysine hydrobromide (PDL), PreScission^®^ Protease, SYPRO^®^ Orange Protein Gel Stain and Thrombin Protease were from Sigma-Aldrich (Dorset, UK). 5,7-Dibromo-6-fluoro-3,4-dihydro-2-methyl-1(2H)-quinolinecarboxaldehyde (CE3F4) was from Tocris (Bristol, UK). Alfa Aesar™ Ponceau S, BL21 Star (DE3) One Shot Chemically Competent E.coli, Biotium Covergrip Coverslip Sealant, Bovine Serum Albumin (BSA), DL-1,4-Dithiothreitol (DTT), Dimethyl Sulfoxide (DMSO), EZ-Link™ Sulfo-NHS-LC-Biotin, Isopropyl-β-D-thio-galactopyranoside (IPTG), Lipofectamine 3000 Transfection Reagent, Lipofectamine™ LTX Reagent with PLUS™ Reagent, PageBlue Protein staining solution, Phosphate-Buffered Saline (PBS) Tablets (10 mM sodium phosphate, 2.68 mM potassium chloride, 140 mM sodium chloride, pH 7.45), Prolong Glass Antifade Mountant and SuperSignal™ West Pico chemiluminescent substrate were purchased from Thermo Fisher Scientific (Waltham, MA, USA). 8-(2-[7-Nitro-4-benzofurazanyl]aminoethylthio) adenosine-3′,5′-cyclic monophosphate (8-NBD-cAMP) and 8-pCPT-2′-O-Me-cAMP (D-007) were purchased from Biolog LSI (Bremen, Germany). 5-alpha-Competent E.coli, BL21 (DE3) Competent E.coli, Protein G Agarose Beads, Protein G Magnetic Beads, Prestained protein standard Marker Broad Range (11–190 kDa) and RIPA Buffer (10×) were from New England Biolabs (Hertfordshire, UK). His-Tag (27E8) (Magnetic Bead Conjugate) and His-Tag (27E8) (Sepharose^®^ Bead Conjugate) were purchased from Cell Signaling Technology (Danves), MA, USA). Nitrocellulose membranes were purchased from Bio-Rad Laboratories Ltd. (Hertforshire, UK).

### 2.2. Protein Purification

Colonies from stock plates of transformed E.coli bacteria expressing recombinant EPAC1ΔDEP, EPAC1ΔDEP mutants (L273W, D276R, R279L and F300D; provided by Professor Holger Rehmann, Hochschule Flensburg, Germany), EPAC2ΔDEP, or EPAC1-CNBD were used to inoculate LB-Broth supplemented with 100 µg/mL ampicillin (or kanamycin for EPAC1ΔDEP mutants) and left to grow overnight with shaking (200 RPM) at 37 °C. These pre-cultures were then diluted 1:20 in LB-Broth supplemented with 100 µg/mL appropriate antibiotic and then incubated for a further four hours (37 °C with shaking) until an optical density reading at 600 nm (OD_600_) reached two (Biochrom WPA S1200+ Visible Spectrophometer). To induce the expression of GST-tagged proteins, fresh isopropyl-β-D-thio-galactopyranoside (IPTG) was added to a final concentration of 100 µM and then the cultures were left to grow O/N at 19 °C with shaking. To extract GST-fusion protein, pellets were resuspended in 25 mL of lysis buffer (50mM Tris-HCl, pH 7.5, 150 mM NaCl, 5 mM EDTA, 5% (*v/v*) glycerol) per pellet, supplemented with 0.5 mg/mL lysozyme, 0.1% (*v/v*) Triton-X 100 and cOmplete Protease inhibitor cocktail (1 tablet/50 mL)). Cells were then lysed by sonication (SONICS Vibra-Cell™ VCX 130 Ultrasonic Liquid Processor, SONICS) for three minutes (15 s on, 15 s off) at 65% amplitude on ice. Bulk protein purification was then carried out using a previously devised protocol [35] for batch absorption of GST-tagged protein to pre-equilibrated glutathione Sepharose 4B beads (GS4B). The GST-tag was removed from purified proteins by adding 80 U of thrombin and incubating for four hours at 4 °C in a sealed column. Pefabloc was added to the collected eluate to a final concentration of 1 mM to block further thrombin activity. For further purification of EPAC1 proteins, and to remove any degraded, low-molecular-weight cleaved forms of EPAC1, the combined cleaved protein fractions were loaded onto a gel filtration column (HiLoad 16/600 Superdex 200 pg, ÄKTA, GE Healthcare) equilibrated with GFC buffer (PBS; 150 mM NaCl, 2.5% (*v/v*) glycerol). The fractions of ÄKTA purified EPAC1ΔDEP protein were combined and analyzed by SDS-PAGE and PageBlue staining or Ponceau-S staining. Protein concentrations were determined using A_280_ aliquots were stored at −80 °C.

### 2.3. Biotinylation of EPAC1ΔDEP Protein

Purified EPAC1ΔDEP was biotinylated using amine reactive Biotinylation reagent, EZ-Link™ Sulfo-NHS-LC-biotin (Thermo Fisher Scientific, Waltham, MA, USA) in a 10-fold molar excess for 2 h on ice. Excess biotin was removed by diafiltration into PBS, pH 7.4. Successful biotinylation was confirmed using Western blotting developed with Streptavidin-HRP (ab7403; Abcam, Cambridge, UK).

### 2.4. Phage-Display Screens

Phage screens were performed using a Type II Affimer library, T2[9_9]ph4v1 (Avacta Life Sciences, Wetherby, United Kingdom). Up to three rounds of panning were performed during each screen. During two selections, EPAC1 was immobilized alone (S01, S04) and additional selections were performed with either 10 µM D-007 (EPAC1-agonist) (S02, S05) or 10 µM CE34F (EPAC1-antagonist) (S03, S06) in the buffer (PBS, pH 7.4). All phage selections consisted of three panning rounds. Bound phage were used to infect E.coli cells and then plated. The Affimer protein coding regions from each phage screen were subcloned into a vector containing both HA and His6 tags (pEtLECTRA cHAH6) and transformed into E.coli. 90 positive colonies were taken from pans two and three of each of the six phage selections (S01-S03; S04-S06; 1080 in total). Each colony was grown overnight and the resulting candidate Affimer proteins were purified by immobilized metal affinity chromatography. The concentrations of the purified Affimer proteins were normalized to 2.5 µg/mL for use during primary screening.

### 2.5. Orthogonal Screening of Identified Affimer Clones

HA-tagged Affimers from the 90 selected clones from phage-display screens were screened in multiple independent bead-based multiplex binding assays using a flow cytometer Intellicyt iQue instrument to confirm their interaction with biotinylated EPAC1ΔDEP. The iQue screening platform is a bead-based flow system for quick analysis of clones against a multiplexed target set. Biotinylated EPAC1ΔDEP and mIgG2b (positive control) were immobilized to streptavidin-functionalized beads, in the presence of 10 µM D-007 (EPAC1-agonist) or 10 µM CE34F (EPAC1-antagonist) in the final assay solution. The HA-tagged Affimers interacting with bead-bound EPAC1ΔDEP were detected using anti HA-tag antibodies with an AlexaFluor 488 conjugate. Similarly, the immobilized mIgG2b positive control was detected with and anti-mIgG2b HA tagged Affimer (G12) followed by Alexa-fluor-conjugated anti-HA-tag antibodies. Affimers found to interact with immobilized EPAC1ΔDEP were further tested for cross-reactivity by testing their interaction with bead-immobilized human and dog C-reactive protein (CRP) and/or anti-carcinoembryonic antigen (CEA). Affimers displaying no cross-reactivity were sequenced and used in follow-on assays.

### 2.6. Dot Blotting

Different concentrations of recombinant EPAC1ΔDEP (GST-tag free) were spotted onto nitrocellulose membranes. Antibody/Affimer interactions were then detected by far-Western blotting. The membranes were blocked for one hour at RT with 5% (*w/v*) “Marvel” milk (antibody blot) or BSA (Affimer blot) in 1 × TBST followed by a two-hour incubation at RT in either 4.5 nM (1:1000) of 5D3 antibody in 5% (*w/v*) milk in 1 × TBST, or 45 nM of Affimer diluted in 5% (*w/v*) BSA in 1 × TBST. Membranes were then incubated for one hour at RT with an appropriate secondary antibody (anti-mouse for 5D3 antibody or His-tag for Affimer) and the protein signal was detected with ECL (SuperSignal™ West Pico chemiluminescent substrate; Thermo Fisher Scientific, Waltham, MA, USA) and visualized using a Fusion FX imaging system.

### 2.7. Microscale Thermophoresis (MST)

The Monolith NT™ Protein Labelling Kit Red-NHS containing the red fluorescent dye NT-647 was used to label recombinant EPAC1-CNBD protein according to the manufacturer’s protocol. Equal volumes of labelled protein were then added to Monolith NT.115 capillaries (Nanotemper technologies) and then measured at LED/excitation set at 80–100% and MST power of 60% (high setting) on the MST instrument. The normalized fluorescence (Fnorm (%)) from MO.Affinity Analysis software version 2.2.4 (Nanotemper technologies) was normalized as a fold change with the relative fluorescence associated with the lowest concentration of antibody set to 1 (Arbitrary Units: AU). The pEC50 was determined by nonlinear curve fitting analysis (Log (agonist) vs. response (three parameters)) GraphPad Prism.

### 2.8. Protein Interaction ELISA Assays

GST-tagged recombinant proteins (1 µg/100 µL/well) were added to Pierce™ glutathione coated plates (15,140; Thermo Fisher Scientific), and incubated overnight at 4 °C. 100 µL/well of 0.002–0.250 µM 5D3 or 5B1 antibody, or 0.004–4 µM of Affimer was then added and incubated for one hour at RT. 100 µL/well of 1:10,000 anti-mouse or anti-his-tag HRP-conjugated secondary antibodies were then added to 5D3/5B1 antibody or Affimer wells, respectively, and incubated for a further one hour at RT. Recombinant protein alone was used as a control. Each experiment was performed in triplicates and between each incubation step, the wells were washed three times with wash buffer (10 mM Tris-HCl pH 7.4, 150 mM NaCl and 0.05% (*v/v*) Tween-20 buffer). Protein, antibodies and Affimers were diluted in assay buffer (10 mM Tris-HCl pH 7.4 and 150 mM NaCl buffer). Plates were kept covered during all incubation steps. Finally, 100 µL/well of 3, 3′,5,5′-Tetramethylbenzidine Liquid (ELISA) Substrate, Supersensitive was added to each well and incubated for 30 min at RT. Detection of color change was measured at wavelength 655 nm using a FLUOstar Omega Microplate reader (BMG Labtech, Aylesbury, Bucks, UK). All data were normalized as a fold change where the lowest concentration of antibody or Affimer was set to 1 (arbitrary units).

### 2.9. Thermal Shift Assays (TSA)

Thermal shift assays were carried out in Thermo Fisher Scientific™ Abgene 1.2 mL Polypropylene 96-well storage plates (10243223; Thermo Fisher Scientific, Waltham, MA, USA), using a modified protocol based on the methods described by Huynh and Partch [36]. All dilutions were prepared in assay buffer (50 mM Tris-HCl pH 7.5, 50 mM NaCl, 2.5% (*v/v*) glycerol and 5 mM DTT). 1–2 µg/well of GST-tagged recombinant protein was combined with 20 × SYPRO Orange dye and added to wells. Plates were sealed with a sheet of optically clear adhesive (MicroAmp™ Optical Adhesive Film; Applied Biosystems) and mixed for 15 min on an orbital shaker at RT before incubating overnight at 4 °C. The fluorescence readout of SYPRO Orange dye was then measured using an Applied Biosystems StepOnePlus Real-time PCR Instrument (System Version 2.2.3; Thermo Fisher Scientific) with excitation wavelength 470 nm and emission wavelength 570 nm over a temperature range from 11 to 80 °C ramped in 0.5 °C increments with plateau times of 30 s. The melting temperature (Tm) (°C) was derived from calculating the first derivative of the fluorescence emission as a function of temperature (−dF/dT) (Equation (1)) where Tm is the lowest first derivative value. For multiple experimental replicates, the mean Tm was calculated with standard error of the mean.

Equation (1). First Derivative Calculations.

dF = F (fluorescence emission) − T (temperature) dT = Tx − Tx (e.g., T2 − T1) −dF/dT = −(dF ÷ dT)(1)

### 2.10. Cell Culture

COS1 cells were grown in Dulbecco’s modified Eagle’s medium (without added glutamine) (DMEM), 10% (*v/v*) fetal bovine serum, 2 mM glutaMAX, and 100 units/mL and 100 μg/mL penicillin and streptomycin, respectively. Cultures were incubated at 37 °C, 5% (*v/v*) CO_2_. U20S cell lines stably expressing pBabe-Flag-Epac1 (HS 1-881) (EPAC1 U2OS), pBabe-Flag-Epac2 (EPAC2 U2OS) or vector alone (Control U2OS), were gifts from Professor Holger Rehmann (Hochschule Flensburg, Germany). U2OS cells were grown in Dulbecco’s modified Eagle’s medium (without added glutamine) (DMEM), 10% (*v/v*) fetal bovine serum, 2 mM glutaMAX, and 100 units/mL and 100 μg/mL penicillin and streptomycin, respectively and incubated at 37 °C, 5% (*v/v*) CO_2_. Puromycin (2 mg/L (*w/v*)) was added to growth medium to maintain stable expression of EPAC1 and EPAC2 U2OS cell lines only.

### 2.11. SDS-PAGE and Immunoblotting

Equal amounts of recombinant proteins or cell lysates were separated on 8, 10, 12 or 12.5% (*w/v*) SDS-PAGE gels, depending on protein size, by electrophoresis. Lysates were then wet transferred from gels to nitrocellulose membranes using Mini Trans-Blot^®^ electrophoretic transfer cell (Bio-Rad) and 1x transfer buffer (250 mM Tris Base, 1.92 M Glycine) for one hour and 30 min at 80 V. Membranes were then blocked in 5% (*w/v*) “Marvel” milk powder in 1 × TBST (100 mM Tris-HCl, pH 7.4, 59 mM NaCl, 0.1% (*w/v*) Tween-20) for one hour at RT. Primary antibodies diluted in 1 × TBST were incubated overnight at 4 °C followed by a one hour incubation at RT with the secondary antibody diluted in 1 × TBST. Protein signals were detected using ECL (SuperSignal™ West Pico chemiluminescent substrate) and visualized using Fusion FX imaging software (Vilber Lourmat, France).

### 2.12. Immunoprecipitation (IP)

U2OS cells were grown to 100% confluency in 6-well plates (9.6 cm^2^ per well) and then placed on ice and washed with 1 mL/well ice-cold PBS. Cells were then lysed with 0.5 mL/well lysis buffer (1x RIPA buffer supplemented with 1 × complete Protease inhibitor cocktail). Cell lysates were subjected to immunoblotting with 5D3 mAb (1.35 or 2.6 µg), 5B1 mAb (2.5 µg), rabbit IgG (4 µg) antibodies or unbound Affimer either alone (2.6 µg) and incubated with rotation for 30 min at 4 °C. Protein G Agarose or α-His Tag Sepharose^®^ beads were then added and incubated with rotation for one hour at 4 °C. Beads were then collected by centrifugation and heated in 1x sample buffer for five minutes at 95 °C. The input and immunoprecipitated samples were then analyzed by Western blotting.

### 2.13. Immunofluorescent Confocal Microscopy

COS1 cells were seeded to at least 90% confluency onto PDL-coated 25 mm diameter No 1.5H high-precision glass coverslips and allowed to adhere overnight in growth medium (37 °C, 5% (*v/v*) CO_2_). Cells were then transiently co-transfected with 780A-mCherry DNA and EPAC1-FLAG or EPAC1-(K212R)-FLAG. Cells were then transfected with X-tremeGENE 360 (XTG360) Transfection Reagent (Sigma-Aldrich) as per the manufacturer’s protocols. To check the transfection efficiency wide-field imaging was carried out on FLoid™ Cell Imaging Station (Thermo Fisher Scientific 4471136), normal white light and red or green fluorescence were used. After 48 h, growth medium containing transfection reagents was replaced with fresh medium and coverslips were incubated for 30 min at 37 °C, 5% (*v/v*) CO_2_. Cells were then stimulated or unstimulated with 100 µM of 007 and incubated for 15 min at 37 °C, 5% (*v/v*) CO_2_ before fixation with 2 mL/well of 4% (*v/v*) paraformaldehyde (prepared in PBS) for 15 min at RT. Cells were then quenched to remove free aldehydes with 2 mL/well of filter sterilized (Whatman Puradisc 25 mm syringe filter 0.2 µm) 50 mM NH_4_Cl (prepared in PBS) for 10 min at RT. Cells were then permeabilized with 2 mL/well of 0.1% (*v/v*) Triton-x 100 in PBS for 10 min at RT. Cells were washed three times with PBS between each incubation. Cells were then blocked for one hour at RT in blocking buffer (1% (*w/v*) BSA, 0.3% (*v/v*) Triton-X 100 in PBS, filtered (Whatman Puradisc 25 mm syringe filter 0.2 µm)). Primary and secondary antibodies (α-FLAG and goat α-mouse IgG H + L (Alexa Fluor 488)) were prepared in blocking buffer and cells were incubated for one hour at RT. Coverslips were mounted onto Fisherbrand™ microscopic glass slides (12383118; Thermo Fisher Scientific) using Prolong™ Glass Antifade Mountant, sealed with Biotium covergrip coverslip sealant and stored at 4 °C. Samples were analyzed using a Leica HC PL APO C52 63x water objective on a Leica TCS SP8 STED 3 × confocal microscope. Samples were excited using a Supercontinuum White Light Laser at 488 and 580 nm for Alexa Fluor 488 and mCherry, respectively, using a Leica HyD1 hybrid detector with detection windows of 500–550 nm for Alexa Fluor 488 and 590–650 nm for mCherry.

### 2.14. Data Analysis

Calculations were performed using Microsoft Excel 2016 for Windows (Microsoft Software) and graphs were made using GraphPad Prism version 5.2 or 8.4.2 for Windows (GraphPad Software LLC, San Diego, CA, USA). Unless otherwise stated, data values are represented as the mean ± standard error of the mean (SEM) or the mean ± standard deviation (SD). The density of protein bands from Western blots was quantified using ImageJ/Fiji version 1.53c (National Institute of Health, Bethesda, Maryland, USA).

To compare two sets of means with a minimum of three experimental repeats, *p*-values were obtained from unpaired *t*-tests using GraphPad Prism version 5.2/9.0.0 for Windows. Analysis of variance (ANOVA) was used for data sets with three or more sets of means, with a minimum of three experimental repeats. *p*-values were from one-way ANOVA with Dunnett’s multiple comparison test using GraphPad Prism versions 5.2/9.0.0.

Confocal images were acquired using the Leica Application Suit X software version 3.0.15 and saved in the LIF format and analyzed using ImageJ/Fiji. Z-stacks were taken with a zoom factor of 1.3 (2048 × 2048 nm). Deconvolution was performed using Huygens Professional software using default settings apart from 0.01% quality change threshold and images were saved in the ICS image format and analyzed using ImajeJ/Fiji. Scale bars were added to microscopy images using the ImageJ Microscopy Scale plugin (National Institute of Health, Bethesda, Maryland, USA). Using ImageJ/Fiji, Z-stacks were analyzed using the Stacks Z-project maximum intensity projection method. Brightness and contrast adjustments post-acquisition were performed using ImageJ/Fiji.

## 3. Results

### 3.1. Phage-Display Selection Using EPAC1ΔDEP as a Target for Affimer Production

**Figure 1:**
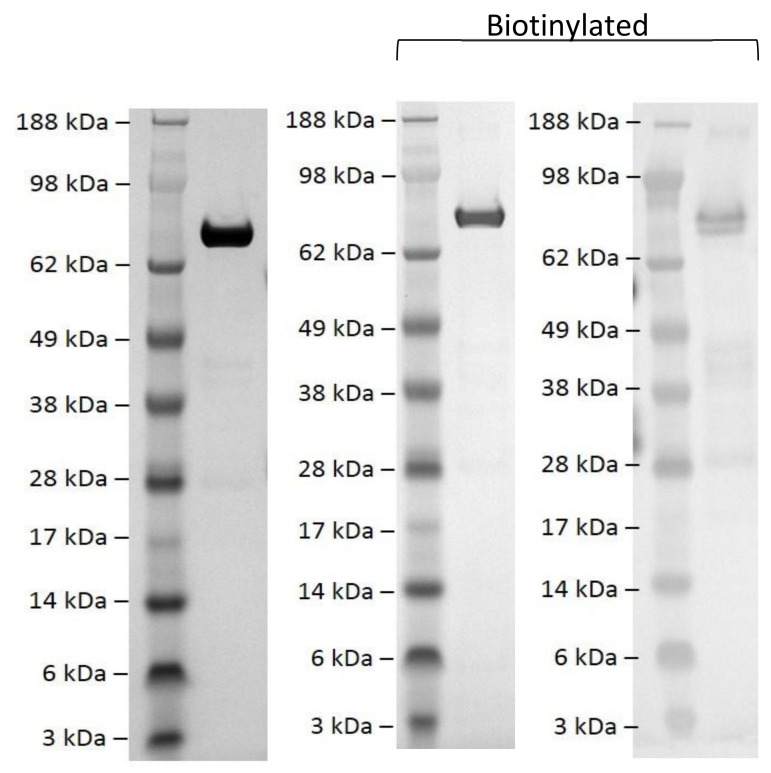
Biotinylation of EPAC1ΔDEP protein. EPAC1ΔDEP-GST protein was expressed and purified from E.coli and the GST removed by limited proteolysis (left panel). EPAC1ΔDEP was then biotinylated as confirmed by SDS-PAGE and Coomassie staining (center panel) and Western blotting (right panel). SeeBlue Plus2 protein markers were used for size comparison.

**Figure 2:**
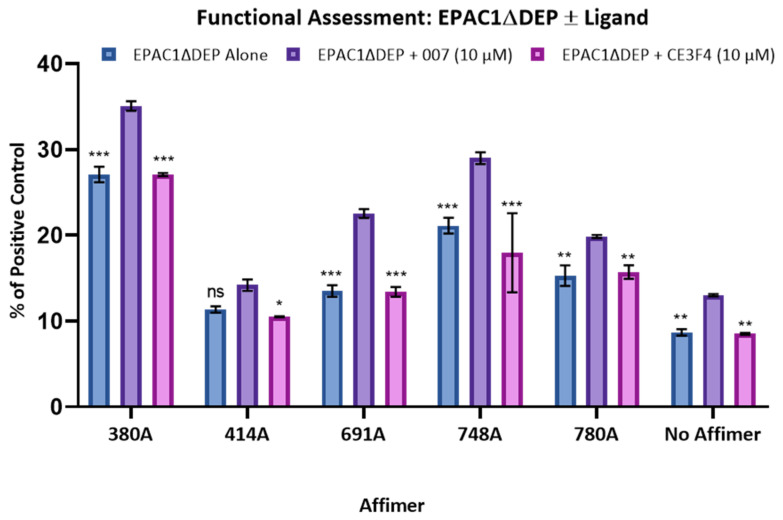
Assessment of Affimers binding to EPAC1ΔDEP in the presence or absence of agonist and antagonist. Affimer binding to biotinylated EPAC1ΔDEP protein was determined by iQue assay, in the presence and absence of 10 µM 007 (EPAC1 agonist) or 10 µM CE3F4 (EPAC1 antagonist). Data were normalized to the median fluorescent intensity of the positive control Affimer (Affimer G12 against immobilized mIgG2b), which was set to 100%. Significant increases in binding were determined by one-way ANOVA. Relative differences in binding relative to EPAC1ΔDEP plus 10 µM 007, are indicated; * *p* < 0.05; ** *p* < 0.001 and *** *p* < 0.0001, respectively (*n* = 3). Results are presented as the means ± SD (*n* = 3). ns: Not significant.

In order to generate Afimers against EPAC1, we purified recombinant EPAC1ΔDEP to homogeneity, based on a previously devised protocol [36]. EPAC1ΔDEP was then biotinylated using an amine-reactive biotinylation reagent as described in Materials and Methods (Figure 1). To identify EPAC1-directed Affimers that bind selectively to either the active form (agonist 007-bound), inactive form (antagonist CE3F4-bound) or the ligand-free forms of EPAC1ΔDEP, six separate in vitro phage-display screens against biotinylated EPAC1ΔDEP were carried out using Avacta’s standard double loop type II library (Table 1). From these screens, 9 positive binders were identified that interacted with biotinylated EPAC1ΔDEP, irrespective of the presence or absence of the EPAC1 agaonist, 007, or the EPAC1 antagonist, CE3F4. To confirm positive interaction with EPAC1ΔDEP, the nine Affimers, identified from the phage-display screens, were re-screened using a bead-based, fluorescent assay (iQue assays, Figure 2). From these assays, five strong Affimer binders were identified, 380A, 414A, 691A, 748A and 780A, which showed a significant increase in binding to EPAC1ΔDEP, compared to no Affimer control (Figure 2). Affimer 380A was identified as being consistently the strongest EPAC1ΔDEP-selective Affimer binder, while 414A was consistently the weakest of the five (Figure 2). There was a significant increase in binding of all five Affimers to EPAC1ΔDEP in the presence of 007 but no significant differences in binding were observed in the presence of CE3F4 (Figure 2). Therefore, through the functional assessment of EPAC1ΔDEP-selective Affimers identified from the phage-display screens detailed in Table 1, Affimers with weak affinities and/or cross-reactivity negative control proteins were deselected. This gave rise to five unique Affimers, 380A, 414A, 691A, 748A and 780A, that were identified as selective binders for EPAC1ΔDEP and whose interaction was enhanced in iQue assay in the presence of the EPAC1 agonist, 007.

### 3.2. In Vitro Interaction of Isolated Affimers with EPAC1ΔDEP

**Figure 3:**
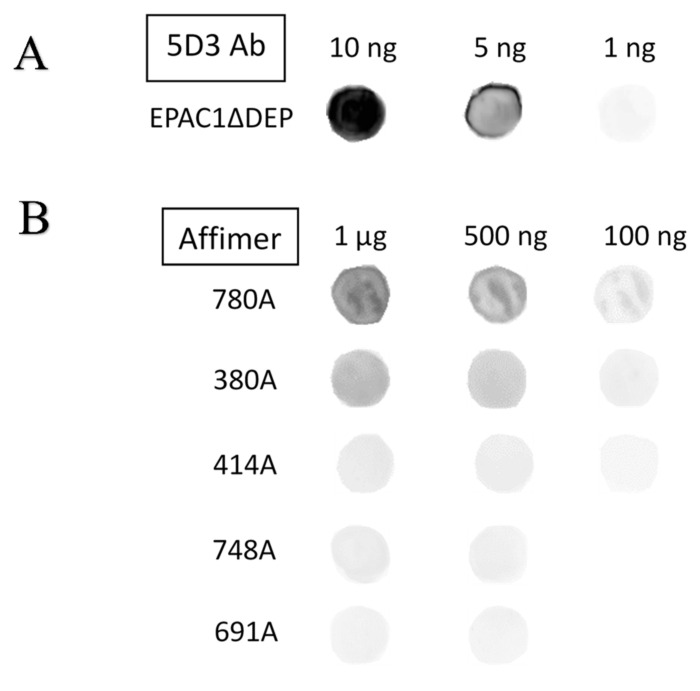
Dot blotting of anti-EPAC1 antibody and Affimer binding to recombinant EPAC1ΔDEP protein. Recombinant EPAC1ΔDEP protein was spotted onto nitrocellulose membranes as indicated. Membranes were then blocked for one hour in 5% (*w/v*) milk or BSA in TBST followed by two hours incubation with either (**A**) 4.5 nM of anti-EPAC1-antibody (mAb 5D3) in 5% (*w/v*) milk in TBST or (**B**) 45 nM of Affimer (780A, 380A, 414A, 691A and 748A) in 5% (***w/v***) in TBST. Membranes were then incubated for one hour with either anti-mouse IgG-HRP, for mAb 5D3, or anti-His-tag HRP, for Affimer, secondary antibodies. All incubations were performed at room temperature. Protein signals were detected by ECL and visualized by Fusion FX imaging software. The image is representative of an experiment carried out on three separate occasions.

To confirm interaction of identified Affimers with non-biotinylated EPAC1 in solid phase assays, 1–10 ng or 0.1–1 µg of recombinant EPAC1ΔDEP protein were spotted onto nitrocellulose membranes and then incubated with either the EPAC1-selective antibody mAb 5D3 or the test Affimers from Figure 2 and then probed with HRP-conjugated anti-mouse or anti-His tag secondary antibodies, respectively. Protein–antibody/Affimer interactions were then detected by ECL. From these dot blots, it could be seen that the EPAC1 antibody was able to detect all three concentrations of EPAC1ΔDEP protein (Figure 3) and, as EPAC1ΔDEP protein concentration decreased, so did the antibody signal (Figure 3).

In terms of Affimer binding, the top three binders, in descending order, were 780A, 380A and 414A, since they showed distinct binding to EPAC1ΔDEP protein at all three concentrations (Figure 3). 748A and 691A, in contrast, were very weak binders and were unable to detect 100 ng of protein (Figure 3). Ultimately, the EPAC1-specific antibody, mAb 5D3, appears to be a better binder to recombinant EPAC1ΔDEP in this assay as protein signal was much higher at only 10 ng of both recombinant proteins in contrast to 780A signal when bound to 1 µg of EPAC1ΔDEP protein (Figure 3). Nevertheless, from the dot blot results, the top three Affimer binders to EPAC1ΔDEP were identified as being 780A, 380A and 414A, with Affimer 780A representing the top binder.

**Figure 4:**
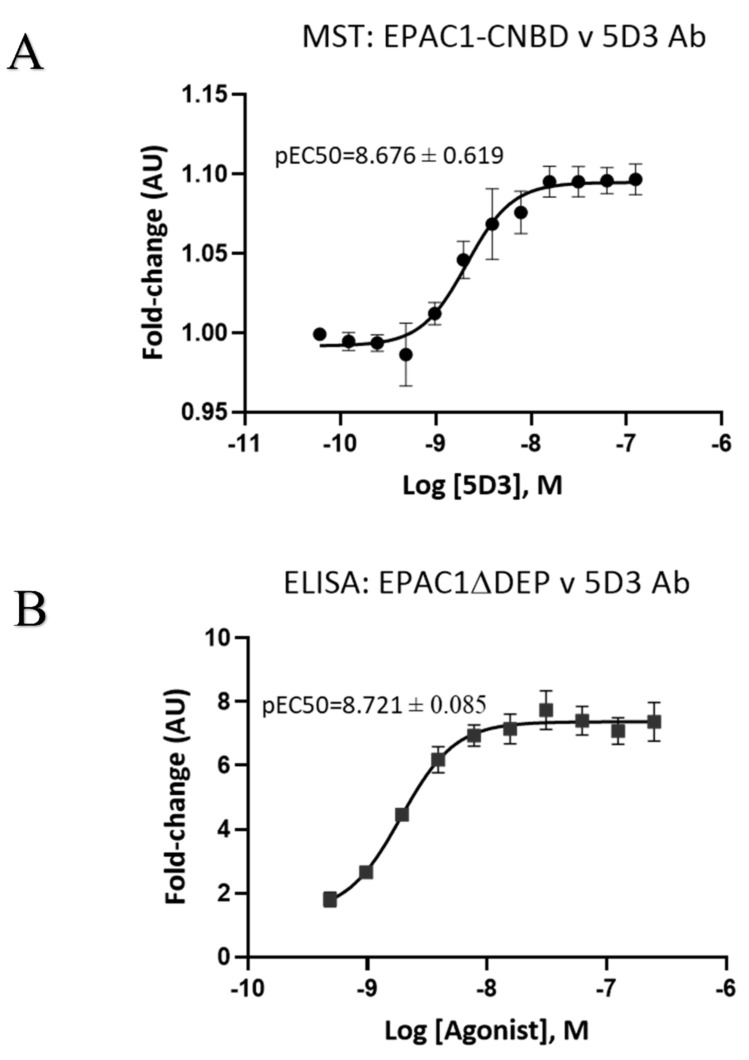
Relative binding affinities of anti-EPAC1 antibody (mAb 5D3) to recombinant EPAC1-CNBD or EPAC1ΔDEP using microscale thermophoresis (MST) and ELISA. (**A**) Recombinant EPAC1-CNBD protein was labelled for MST as described in Materials and Methods using the Monolith NT™ Protein Labelling Kit RED-NHS and then diluted to 20 nM with assay buffer. A serial dilution of mAb 5D3, ranging from 0.03 to 125 nM, was also prepared in assay buffer, and then added to 16 capillary tubes containing equal volumes of target protein. Samples were then measured by Monolith NT1.15 (Nanotemper) at LED/excitation set to 80–100% and MST power of 60% (high setting). (**B**) A 1 µg/well of recombinant EPAC1ΔDEP protein added to glutathione-coated plates and incubated overnight at 4 °C. Serial dilution of 5D3 Ab (0.2–125 nM) was prepared in assay buffer and incubated for 1 h at RT. Anti-mouse antibody (1:10,000) was then added and incubated for a further 1 h at room temperature. The ELISA substrate was added and absorbance measurements at 655 nm were taken after a 30 min incubation at room temperature using a FLUOstar Omega Microplate reader. The experiments were performed in triplicates. Data for both MST and ELISA were normalized as a fold change with the relative fluorescence associated with the lowest concentration of antibody set to 1 (Arbitrary Units (AU)). Error bars are representative of the standard error of the mean (*n* = 4) and pEC50 (mean ± SEM) was determined by using Log (agonist) vs. response-variable slope (four parameters) regression curve fitting (Graphpad Prism).

**Figure 5:**
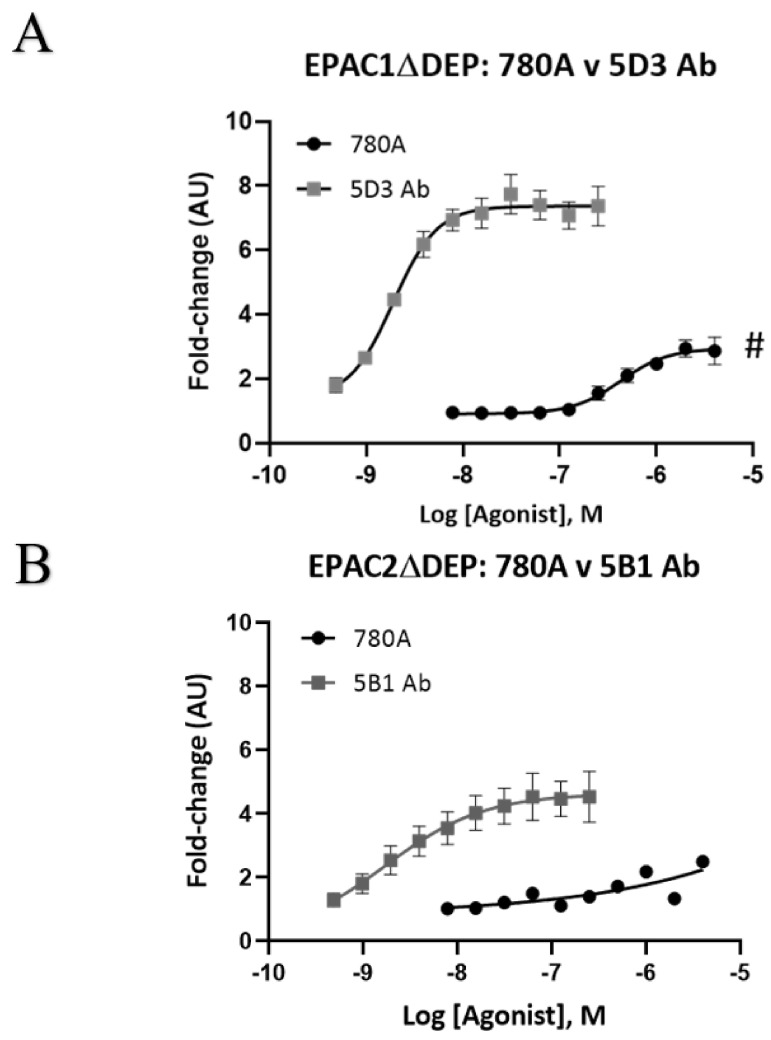
Relative binding affinities of Affimer 780A versus anti-EPAC1- (mAb 5D3) and anti-EPAC2-selective (mAB 5B1) antibodies for recombinant EPAC1ΔDEP and EPAC2 ΔDEP proteins. For each ELISA, 1 µg/well of GST-tagged recombinant protein, either A) EPAC1ΔDEP or B) EPAC2ΔDEP was added to glutathione-coated plates and incubated overnight at 4 °C. Serial dilutions of His-tagged Affimer (780A) (0.004–4 µM) or antibodies (**A**) mAb 5D3 and (**B**) mAb 5B1 (0.2–125 nM) were then incubated for one hour at RT. Anti-His-tag or anti-mouse antibodies (1:10,000) were then added to the Affimer or antibody-containing wells, respectively, and incubated for a further one hour at room temperature. The ELISA substrate was then added and absorbance measurements at 655 nm were taken using a FLUOstar Omega Microplate reader. The experiments were performed in triplicates. Data were normalized as a fold change with the relative fluorescence associated with the lowest concentration of Affimer or antibody being set to 1 (Arbitrary Units (AU)). Error bars are representative of the standard error of the mean (*n* = 1–5) and the Log (agonist) vs. response—variable slope (four parameters) nonlinear regression curve fitting was used to obtain pEC50 (GraphPad Prism). A significant decrease in binding efficacy (Emax) relative to EPAC1ΔDEP plus 5D3 Ab (^#^ *p* < 0.0005) was determined by unpaired *t*-test (GraphPad Prism).

Having determined that 780A is the most effective binder to EPAC1ΔDEP of the Affimers tested, protein interaction ELISA assays were used to determine the relative affinity of 780A for interaction with recombinant EPAC1ΔDEP and EPAC2ΔDEP (Figure 5). We first validated that protein ELISAs were suitable for affinity measurements by comparing the binding affinities of the commercially available EPAC1 antibody (mAb 5D3) to recombinant EPAC1 proteins by comparing MST results with and ELISA results and found that both techniques gave comparable pEC50 values (Figure 4) for the binding of 5D3 to the EPAC1 CNBD (MST) and EPAC1ΔDEP (ELISA). Similarly, Affimer 380A binding to the EPAC1 CNBD gave similar results, in terms of relative EC50s, using both MST and ELISA approaches. Due to this, we decided to use the ELISA approach in subsequent experiments due to problems with labelled, full-length EPAC1ΔDEP aggregating at high concentrations in MST experiments.

We therefore used ELISA assays to compare 780A binding with mAb 5D3 and a commercially available EPAC2-specific antibody (mAb 5B1) as positive controls (Figure 5). For this, GST-tagged forms of recombinant EPAC1ΔDEP and EPAC2ΔDEP were immobilized to GSH-coated plates and then incubated with various concentrations of Affimer 780A or mAbs. From these dose–response curves, the pEC50 values for mAb 5D3 and Affimer 780A binding to EPAC1ΔDEP protein can be determined (Table 2). Although saturation of the binding of 780A to EPAC2ΔDEP could not be determined, a pEC50 of approximately 9 was obtained for mAb 5B1 binding to this protein (Table 2). With a pEC50 of approximately 9, mAB 5D3 was a significantly (*p* < 0.05) more effective EPAC1ΔDEP binder than 780A which had a pEC50 of approximately 6 (Table 2). In addition, from ELISA results, Affimer 780A was identified to have a higher affinity for EPAC1-CNBD over Affimer 380A (Table 2). Moreover, Affimer 780A bound to both recombinant EPAC1ΔDEP and EPAC1-CNBD proteins but did not bind to either recombinant form of the EPAC2 protein.

### 3.3. Mapping of Affimer Interaction Sites on EPAC1

**Figure 6:**
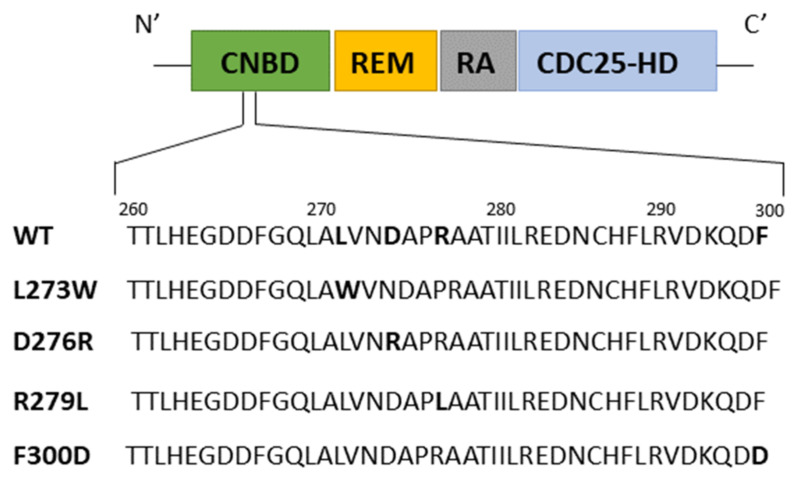
EPAC1ΔDEP structure and sequence alignment. Domain organization of recombinant EPAC1ΔDEP. CNBD, cyclic nucleotide binding domain; REM, Ras exchange motif; RA, Ras association domain; CDC25-HD, CDC25-homology domain. A sequence alignment of the region in the CNBD (amino acids 260–300) from wild-type (WT) EPAC1ΔDEP with the four mutant forms of the protein shown. Original and mutated residues are highlighted in bold in each sequence.

**Figure 7:**
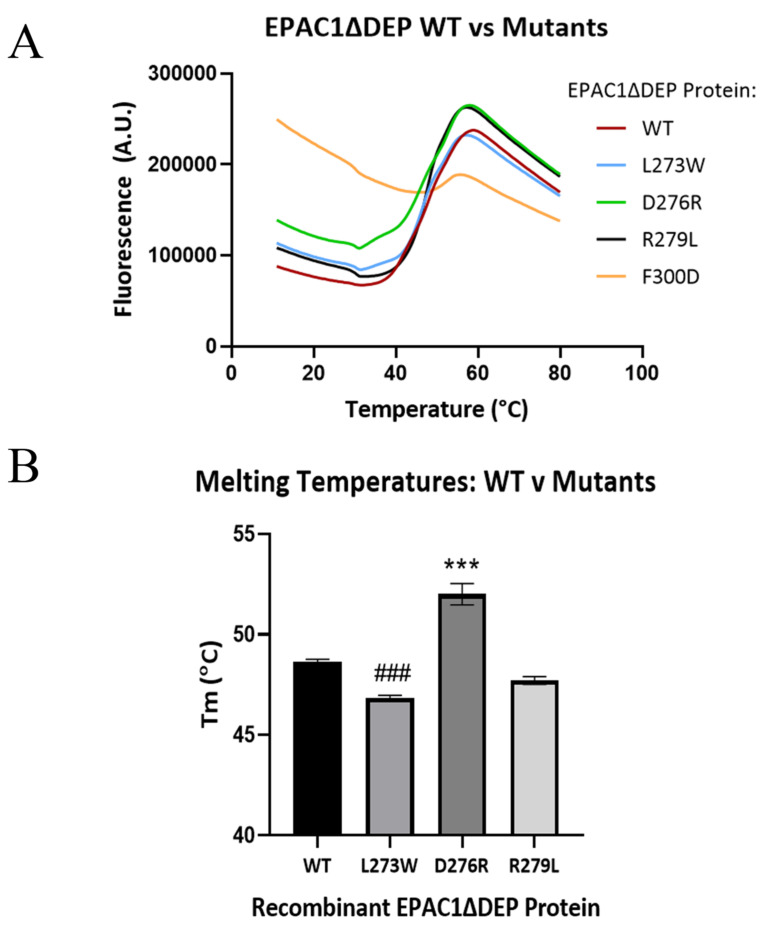
Thermal shift profiles of recombinant wild-type EPAC1ΔDEP in comparison to mutant EPAC1ΔDEP proteins. Thermal shift assays were performed using 1 ug of recombinant EPAC1ΔDEP wild-type (WT) or mutant (L273W, D276R, R279L, F300D) protein per well. Plates were incubated overnight at 4 °C. with SYPRO Orange dye and then (**A**) fluorescence readouts were measured using an Applied Biosystems StepOnePlus Real-time PCR Instrument set at excitation wavelength 470 nm and emission wavelength of 570 nm over a temperature range from 11 to 80 °C ramped to 0.5 °C increments with plateau times of 30 s. Measurements were performed in duplicates (*n* = 16–23). (**B**) Melting temperatures (Tm ± SEM °C) were obtained by calculating the first derivatives of the fluorescence emission as a function of temperature (−dF/dT). A significant decrease (^###^ *p* < 0.0001) or increase (*** *p* < 0.0001) in Tm relative to WT protein was determined by one-way ANOVA (Graphpad Prism) (*n* = 16–23).

In order to identify where Affimer 780A binds on EPAC1, ELISAs were performed using four EPAC1ΔDEP point mutants, L273W, D276R, R279L and F300D (Figure 6), which had previously been identified as being important for mAb 5D3 binding to the CNBD of EPAC1 [31]. Before proceeding with protein interaction ELISA assays, we first used thermostability assays to determine if the test proteins purified from E.coli were properly folded (Figure 7). Comparative Tms from these results demonstrated that the mutant F300D does not fold properly and that a Tm could not be calculated for this mutant. Mutant F300D was therefore excluded from further protein interaction assays. Although conformational changes were also apparent for mutants L273W and D276R, in comparison to WT EPAC1ΔDEP (Figure 7), these proteins appeared to fold correctly. Mutant R279L showed a comparable Tm to WT EPAC1ΔDEP (Figure 7) and was therefore judged to be correctly folded.

**Figure 8:**
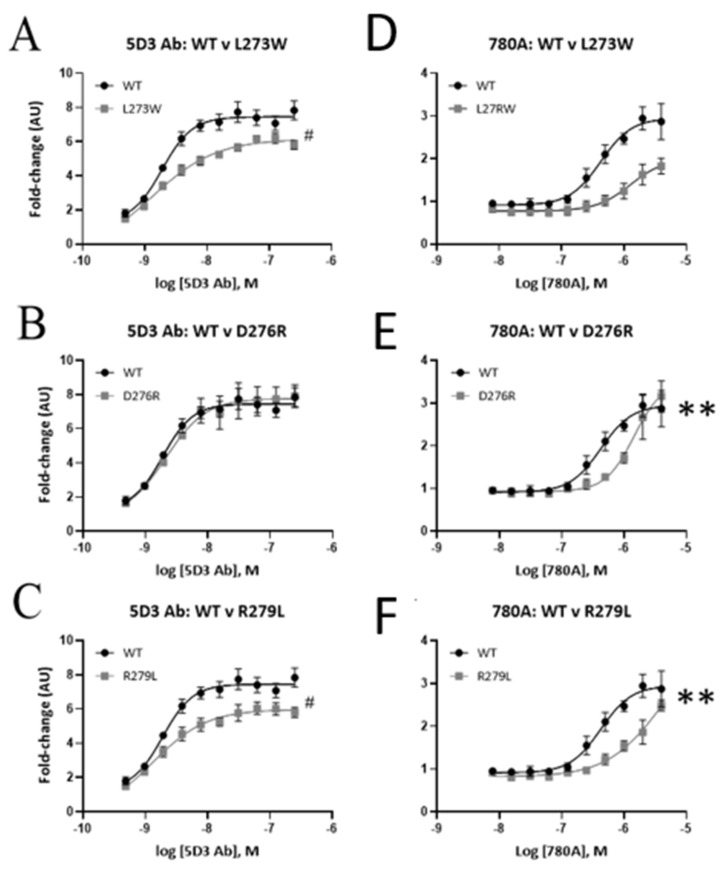
Comparison of the relative binding affinities of Affimer 780A to mAb 5D3 to recombinant EPAC1ΔDEP WT and mutant proteins. GST-tagged recombinant (wild-type (WT) or mutant) protein were added to glutathione-coated plates and incubated overnight at 4 °C. Serial dilutions of antibody (**A–C**) mAb 5D3 (0.2–125 nM) and (**D**–**F**) Affimer 780A (0.004–4 µM) and incubated for one hour at RT. Anti-His-tag or anti-mouse antibodies (1:10,000) were then added and incubated for a further one hour at RT. The ELISA substrate was then added, and absorbance measurements (655 nm) were taken after 30 min incubation at RT using a FLUOstar Omega Microplate reader. Data are normalized as a fold change with the relative fluorescence associated with the lowest concentration of antibody or Affimer set to 1 (arbitrary units (AU)). Error bars represent standard error of the mean (*n* = 3–5). Significant increases in EC50 (** *p* < 0.005) or decrease in Emax (^#^ *p* < 0.05) relative to EPAC1ΔDEP WT were determined by unpaired *t*-test (GraphPad Prism). (**F**) Competition ELISA assays to check overlap between Affimer 780A and antibody 5D3 binding sites. Affinity reagents were used at concentrations on 145 nM and 2 nM, respectively.

ELISA data for binding of mAb 5D3 and 780A to the mutant forms of EPAC1ΔDEP in comparison with wild-type EPAC1ΔDEP are shown in Figure 8. Results demonstrated that the binding efficacy (Emax) of mAb 5D3 was significantly reduced (*p* < 0.05) by mutants L273W and R279L in comparison to EPAC1ΔDEP WT, where it dropped from approximately 7.8 ± 0.6 (A.U.) to approximately 5.8 ± 0.3 (A.U.) (Figure 8A,C). In contrast, the binding of Affimer 780A was significantly decreased by mutants L273W, D276R and R279L (Figure 8F). It can therefore be concluded that while both mAb 5D3 and Affimer 780A share overlapping binding sites in the EPAC1 CNBD, the mode of interaction is different and relies on distinct amino acids, notably D276 for Affimer A780A binding. This is confirmed by competition assays, where unlabelled mAb 5D3 is unable to inhibit binding of labelled Affimer 780A to the EPAC1 CNBD (Figure 8F). In fact, there is a small, non-significant potentiation of Affimer 780A binding, which suggests possible cooperativity of binding of the two affinity agents on the CNBD.

### 3.4. Application of Identified Affimers in Immunoprecipitation Experiments

**Figure 9:**
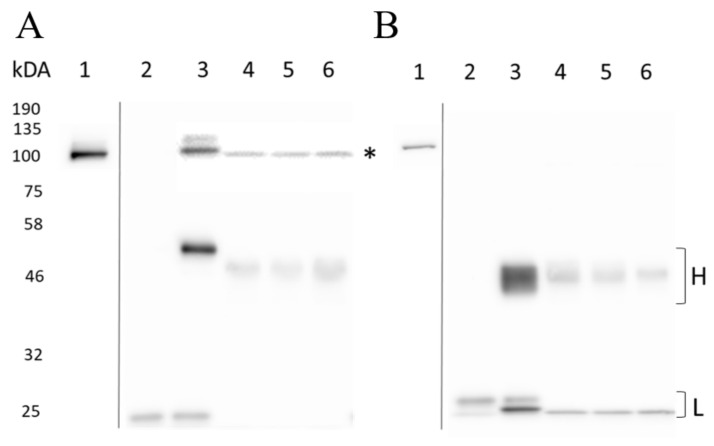
Comparison of antibodies and Affimers for immunoprecipitation of EPAC1 and EPAC2 using. (**A**) EPAC1 and (**B**) EPAC2 U20S cells were harvested in 0.5 mL of RIPA buffer, containing protease inhibitor cocktail, and lysed for 30 min by rotation at 4 °C. Extracts were centrifuged and cell lysates were collected, including inputs (total EPAC protein) (lane 1). To each cell lysate, 4 µg of rabbit IgG antibody (lane 2), 1.4 µg of 5D3 (A: lane 3) or 2.5 µg of 5B1 antibody (B: lane 3), or 2.6 µg of Affimer: 780A (lane 4), 380A (lane 5) and 414A (lane 6) were added and incubated with rotation for one hour (4 °C) before adding 40 µL of Protein G Agarose (lanes 2–4) or α-His-Tag Sepharose^®^ beads (lanes 5–6). After a one-hour incubation at 4 °C, with rotation, antibody-bead complexes were collected while Affimer-bead complexes were collected after an overnight incubation at 4 °C. Beads were then washed five times with 0.5 mL RIPA buffer using centrifugation for the collection of beads. Samples were heated in electrophoresis sample buffer for five minutes at 95 °C and analyzed by Western blotting where 15 and 30 µL of input and isolated immunoprecipitated proteins, respectively, per well from EPAC1 or EPAC2 U20S cells were separated by SDS-PAGE and probed with (**A**) 5D3 antibody or (**B**) 5B1 and α-mouse primary and secondary antibodies, respectively, as described in Materials and Methods. Total EPAC Protein (*), and heavy (H) and light (L) chain bands are indicated. Protein markers (kDa) are also indicated.

To test the ability of the top three EPAC1-selective Affimers, 780A, 380A and 414A to immunoprecipitate (IP) EPAC1 or EPAC2 protein from cells, U2OS cells stably transfected with EPAC1 (EPAC1 U2OS) or EPAC2 (EPAC2 U2OS) were used as a source of cellular EPAC proteins. The performance of these Affimers was compared to EPAC1 specific mAB 5D3 and EPAC2 specific mAb 5B1 (Figure 9). In the immunoprecipitation experiments in Figure 9, EPAC1 or EPAC2 proteins are indicated by an asterisk (*) at an approximately 100 kDa size and total EPAC1 or EPAC2 protein levels can be seen in lane one. The heavy (H) and/or light (L) chains of Protein G Agarose and α-His-Tag Sepharose at approximately 46 and 25 kDa, respectively, could also be observed (Figure 9). In the EPAC1 U2OS cells, it appeared that there were higher levels of EPAC1 protein in comparison to EPAC2 protein in EPAC2 U2OS cells (Figure 9A vs. Figure 9B). As the negative control, IgG Ab did not precipitate any EPAC1 or EPAC2 protein, but the light chain could be seen in lane two, demonstrating that samples were loaded in these lanes (Figure 9).

From these IP results, it could clearly be seen that mAb 5D3 successfully precipitated EPAC1 proteins from cells (Figure 9A), whereas mAb 5B1 was unable to precipitate EPAC2 protein (Figure 9B). Similarly, all three Affimers successfully precipitated EPAC1 protein, as seen in lanes four to six, but did not precipitate EPAC2 protein (Figure 9). The lack of EPAC2 protein in these wells was not due to sample loading error as H and L chains of the α-His-Tag Sepharose could be seen (Figure 9). No differences in precipitated EPAC1 protein levels were observed between the Affimers, but the Affimers did precipitate less EPAC1 protein than mAb 5D3 (Figure 9). Therefore, although Affimers 780A, 380A and 414A continued to show selective interaction with EPAC1 in U2OS cells, mAb 5D3 Ab was still a more potent binder. Nevertheless, all three Affimers showed a selective interaction with EPAC1 over EPAC2, which agrees with the protein interaction ELISA experiments shown above.

### 3.5. Application of EPAC1 Affimers to Confocal Microscopy

**Figure 10:**
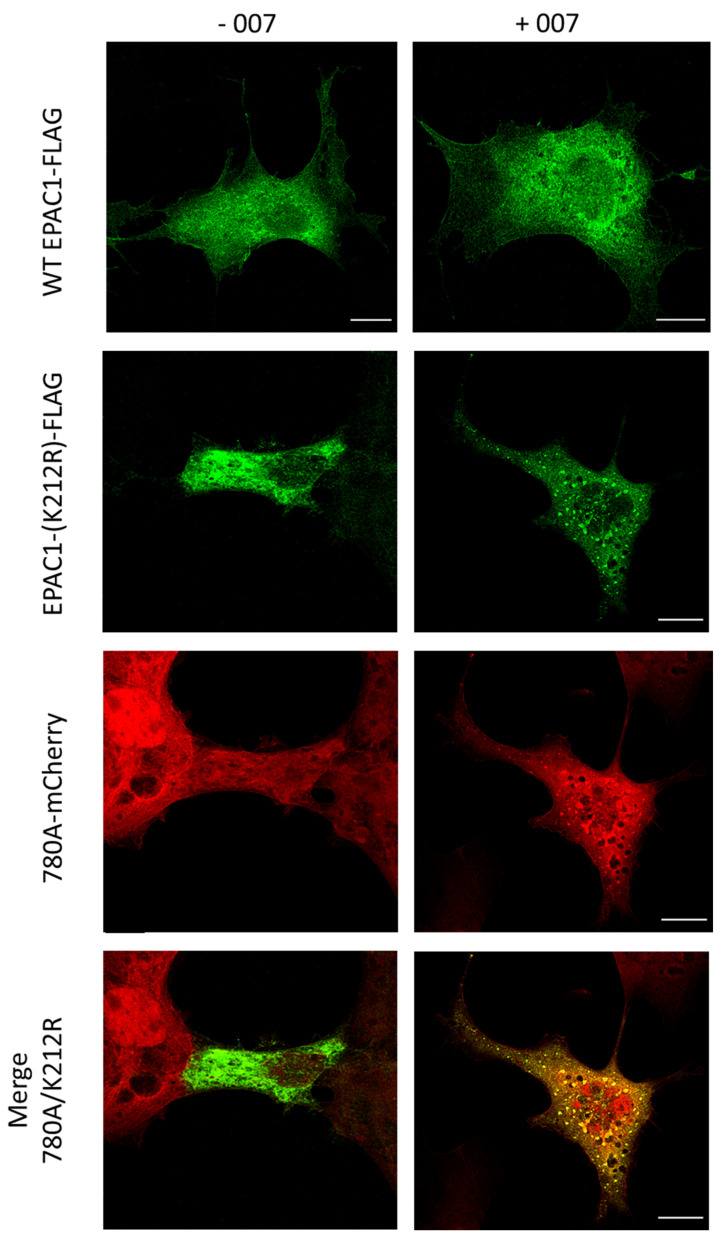
Localization of Affimer 780A-mCherry with co-transfected wild-type and mutant (K212R) EPAC1 in COS1 cells. COS1 cells were transiently co-transfected with 780A-mCherry and WT-EPAC1-FLAG or EPAC1-(K212R)-FLAG 780A-mCherry and then stimulated ± 100 µM of 007 for 15 min (37 °C, 5% (*v/v*) CO_2_). Following fixation, permeabilization and blocking cells were then incubated for one hour each with α-FLAG and goat α-mouse IgG (Alexa Fluor 488 conjugated) primary and secondary antibodies, respectively. Coverslips were then mounted onto glass slides using Prolong™ Glass Antifade Mountant and analyzed on a Leica TCS SP8 STED 3X confocal microscope with a Leica HC PL APO C52 63 × water objective. Samples were excited using a Supercontinuum White laser at 488 and 580 nm for Alexa Fluor 488 and mCherry proteins, respectively, and were detected using a Leica HyD hybrid detector with detection windows of 500–550 nm (Alexa Fluor 488) and 590–650 nm (mCherry). Scale bar: 100 nm.

Having demonstrated that Affimers can interact with cellular EPAC1 in immunoprecipitation experiments, we next asked whether they could be useful reagents for confocal localization experiments in cells. For this, co-transfection experiments were carried out to determine the co-localization of wild-type EPAC1 and Affimer 780A in non-stimulated (-007) and stimulated (+007) COS1 cells. In Figure 10, FLAG-tagged wild-type EPAC1 (WT-EPAC1-FLAG) can be observed in green in the top images and displays a diffuse pattern throughout the cell, which is not affected by 007 treatment. This is very similar to the distribution of mCherry-tagged Affimer 780A-mCherry (780A-mCherry, shown in red) in cells, which is again unaffected by 007 treatment (Figure 9; third pane from the top). Thus, although Affimer 780A and wild-type EPAC1 show similar patterns of distribution throughout the cell, the experiment lacks the resolution to show co-localization of the co-transfected poteins.

To address this, we next examined the localization of Affimer 780A in COS1 cells co- transfected with FLAG-tagged EPAC1 (K212R; Figure 10), which represent a mis-targeting mutant of EPAC1, previously identified in the Baillie laboratory, to obtain improved resolution of any co-localization. We observed that the EPAC1-(K212R) mutant was excluded from the nuclei of both non-stimulated and 007-stimulated COS1 cells as seen in Figure 9. The localization of the EPAC1 mutant was found to be distinctively punctate throughout the cell following stimulation in 007 (Figure 10; second panel from top). In the merged image of a non-stimulated cells, some co-localization of Affimer 780A and the EPAC1-(K212R) mutant was observed in areas out-with the nucleus (merge 780A/K212R left). However, co-localization could be seen more clearly in the stimulated cells in the punctate areas of the cytosol (merge 780A/K212R right). This suggests that Affimer 780A can co-localize with the mistargeted EPAC1-(K212R) mutant in 007-stimulated cells.

## 4. Discussion

To provide a target to generate EPAC1-selective Affimers for phage display, a purification protocol for recombinant EPAC1ΔDEP protein was optimized to obtain protein that was at least 90% pure. In general, the optimized protocol produced approximately 1 mg EPAC1ΔDEP per liter of culture. Purified EPAC1ΔDEP was biotinylated and used as a target in phage-display screens to identify candidate EPAC1-selective Affimers that were subjected to final functional assessment using a fluorescent-based protein interaction screen. Phage-display screens were carried out in the presence of the EPAC-selective agonists, 007, or the antagonist, CE3F4, and sequencing of interacting phage revealed nine unique Affimers, five of which were established as being EPAC1-positive binders in orthogonal screens. Despite the presence of 007 or CE3F4 during phage-display screening, the isolated Affimers did not appear to discriminate between the ligand bound and ligand-free forms of EPAC1.

The binding characteristics of the five Affimers, named 380A, 414A, 604A, 748A and 780A, were further characterized for their ability to interact with EPAC1, using different solid and solution phase in vitro assays. From these assays, Affimers 780A, 380A and 414A were identified using dot blots and ELISA assays as the top three binders that showed selectivity for recombinant EPAC1ΔDEP and EPAC1-CNBD protein, over EPAC2, although their relative binding affinities were much lower than the pre-existing EPAC1 mAb, 5D3. In addition, from these experiments, it was clear that Affimer 780A interacted equally well with the active and inactive conformations of EPAC1, as provoked by ligand binding. Affimer A780 therefore presents technical advantages over mAb 5D3 in that it is not conformation selective, whereas 5D3 favors interaction with the active conformation of EPAC1.

The potential interaction sites of Affimer 780A on EPAC1 were first investigated using ELISA and TSA assays to assess interaction of the Affimer with four CNBD mutants of recombinant EPAC1ΔDEP (L273W, D276R, R279L and F300D), which were originally used to identify the binding epitope for mAb 5D3 in the EPAC1 CNBD [31]. ELISA assays revealed that the binding of Affimer 780A was significantly reduced by D276R and R279L mutants, compared to WT EPAC1, whereas the binding efficacy of mAb 5D3 was significantly impaired by mutants L273W and D276R. Recombinant F300D was found to be in a non-native form by TSA. Thus, although Affimer 780A and mAb 5D3 interact within the same discrete region within the EPAC1 CNBD, it appears that their binding mode are subtly different, that, and the comparative size differences between 780A and mAb 5D3, may go some way to explain the differences in conformational selectivity between the two affinity agents.

Having determined that Affimer 780A is an affective binder for EPAC1, in cell interaction assays were carried out next. Using immunoprecipitation assays, the top three Affimer binders were all found to selectively precipitate EPAC1 protein from U2OS cells expressing EPAC1, but not EPAC2. Moreover, in agreement with in vitro studies, the Affimers did not show preferential interaction with the active form of EPAC1, in comparison with mAb 5D3. Next confocal microscopy experiments were used to visualize the expression of a protein chimera of Affimer 780A with fluorescent mCherry protein in COS1 cells. From these experiments it was found that Affimer 780A localized to the nuclei of COS1 cells, whether or not EPAC1 was pharmacologically activated by treatment of cells with 007. Furthermore, Affimer 780A was observed to co-localize with FLAG-tagged EPAC1 and a mis-targeted mutant of EPAC1, K212R, predominantly at punctate loci throughout the cytosolic regions of COS1 cells, following 007 treatment.

Recombinant protein scaffolds, such as Affimers, can and have been used to observe direct target binding. Affimers are not only small proteins (12–14 kDa), allowing for good penetration of cells, but can also be easily labelled with fluorescent molecules at specific sites [37,38]. This offers improved resolution, providing a more confident assessment of the localization of an Affimer with its specific target, such as EPAC1 in the case of Affimer 780A. Through confocal microscopy studies, Affimer 780A was shown to localize with overexpressed EPAC1, with or without 007 stimulation, further demonstrating that the Affimer does not favor the active or inactive conformations of EPAC1, unlike mAb 5D3, which interacts selectively with active EPAC1 [23]. In addition, the co-localization of Affimer 780A with a mis-targeting EPAC1 mutant (K212R) confirms that the Affimer binds to EPAC1 in cells regardless of location. Furthermore, the subcellular co-localization of the Affimer with EPAC1 predominantly in the nuclear and perinuclear regions here agree with previous studies, using several different cell types, providing further confidence that Affimer 780A interacts with EPAC1 in cells. For example, in COS-7 cells transfected with GFP-tagged EPAC1 protein, fluorescent confocal microscopy showed an association of EPAC1 with the nucleus and along the nuclear membrane [17]. In addition, using an EPAC1 FRET sensor, where EPAC1 was sandwiched between two different fluorescent proteins, active EPAC1 was observed to co-localize to membranes and the cytosol, but particularly at the nuclear envelope and perinuclear region in four different cell lines tested, including HEK293 and NIE-115 neuroblastoma cell lines [39]. Moreover, in an immunocytochemistry (ICC) study, using transfected FLAG-tagged EPAC1 in COS1 cells, the immunoreactivity of EPAC1, was also seen to associate distinctly with the perinuclear region of COS1 cells [18]. Using both live cell fluorescent confocal experiments and FRET imaging of HEK293 cells, it was also observed that active EPAC1 associates with the nuclear envelope, nucleus, PM, endomembranes and cytosol [22]. In this case, however, when cells were stimulated with 007, there was a redistribution of a small subfraction of EPAC1 to the PM [22], which is consistent with what was observed here. Finally, HEK293T cells overexpressing EPAC1-FLAG and immunostained with mAB 5D3 also showed that active EPAC1 associated with the perinuclear region of cells [23]. The fact that the localization of Affimer 780A-mCherry in COS1 cells observed in this project agree with results from the studies described above, suggests that Affimer 780A has the potential to be used as a probe for microscopy studies involving EPAC1.

## 5. Conclusions

Successive phage-display screens were used to identify nine potential EPAC1-selective Affimer candidates, three of which were confirmed as being EPAC1 binders using a range of in vitro assays. From these three, Affimer 780A was identified as being the best EPAC1 binder by dot bot and protein interaction ELISA assays. Little interaction with EPAC2 was observed in these assays. Affimer 780A was found to interact favorably with either the active or inactive conformations of EPAC1. Mutagenesis studies revealed a potential interaction site for Affimer 780A within the CNBD of EPAC1. As such, Affimer 780A was able to interact with and precipitate EPAC1, but not EPAC2, protein from cell extracts and, when tagged with fluorescent mCherry protein, Affimer 780A, was found to co-localize with both wild-type EPAC1 and a non-targeting EPAC1 mutant (K212R), predominantly in the perinuclear and cytosolic regions of cells, respectively. Overall, Affimer 780A represents a first-in-class selective binder for EPAC1 for use in biochemical and cell biology studies of cyclic AMP signaling. Future work will be aimed at improving the affinity of Affimer A780 towards EPAC1 through selective mutation of the Affimer binding site and also generate Affimers to the N- and C-termini of EPAC1. By improving the affinity towards EPAC1 and identifying further interactions sites we hope to extend the usefulness of EPAC1 Affimers for fluorescent microscopy and other applications, including modulation of EPAC1 activity and intracellular targeting.

## Figures and Tables

**Table 1:** Summary of phage-display selection methods. Six different phage-display screens were carried out using biotinylated EPAC1ΔDEP as a target (screens S01–S06). Pre-panning deselection and off-rate deselection was performed in pans 2 and/or 3 as a process of negative selection. Here, homologous proteins, (EPAC1ΔDEP with no ligand) were premixed with the phage library, either as immobilized proteins and/or as proteins in solution. Off- rate deselection involves allowing the Affimer-expressing phage to bind to the target, washing away unbound phage and incubating the phage bound to immobilized target overnight in buffer before eluting the following morning; this drive selection towards Affimer proteins with the slowest off-rates. Enrichment refers to the number of captured phage with affinity for biotinylated EPAC1ΔDEP, as indicated by number of positive, phage-transformed bacteria following the panning step compared to a negative selection well.

Screen	Page Display Method			Phage-Display Enrichment
	Target	Pre-Panning Deselection	Off-Rate Deselection	
S01	EPAC1ΔDEP	None	In pans 2 and 3	9 × over neutravidin
S02	EPAC1ΔDEP plus 007	EPAC1ΔDEP in Pans 2 and 3	In pans 2 and 3	1.2 × over EPAC1ΔDEP
S03	EPAC1ΔDEP plus CE3F4	EPAC1ΔDEP in Pans 2 and 3	In pans 2 and 3	1.2 × over EPAC1ΔDEP
S04	EPAC1ΔDEP	None	In pan 3	~2 × over neutravidin
S05	EPAC1ΔDEP plus 007	EPAC1ΔDEP in Pans 2 and 3	Not performed	2.5 × over EPAC1ΔDEP
S06	EPAC1ΔDEP plus CE3F4	EPAC1ΔDEP in Pans 2 and 3	Not performed	1.4 × over EPAC1ΔDEP

**Table 2:** pEC50 values from ELISA and MST binding assays. The pEC50 (negative log of the EC50) values of either Affimer (780A or 380A) or antibody (mAb 5D3 or mAb 5B1) when binding to different recombinant EPAC proteins were determined by ELISA or MST. The binding of Affimer to EPAC2ΔDEP and EPAC2-CNBD could not be determined because complete confidence interval could not be calculated for at least one parameter of the nonlinear regression curve fitting.

Protein + Affimer/mAb	EPAC1ΔDEP + 780A	EPAC1ΔDEP + mAb 5D3	EPAC1-CNBD + mAb 5D3	EPAC1-CNBD + 780A	EPAC1-CNBD + 380A	EPAC2ΔDEP + mAb 5B1
ELISA pEC50 ± SEM (A.U.)	6.39 ± 0.12	8.72 ± 0.08	-	6.70 ± 0.16	5.52 ± 0.35	8.60 ± 0.01
MST pEC50 ± SEM (A.U.)	-	-	8.68 ± 0.72		6.39 ± 0.24	-
